# Supplementary material for: Sulfide resorption during crustal ascent and degassing of oceanic plateau basalts
Source: Nat Commun. 2019 Jan 8;10:82. doi: 10.1038/s41467-018-08001-3 (PMC6325133; doi:10.1038/s41467-018-08001-3)
Supplement: Supplementary file 1 — Supplementary Information [file 41467_2018_8001_MOESM1_ESM.pdf]

Reekie et al. – Sulfide resorption during crustal ascent and degassing of oceanic plateau basalts

Supplementary Notes

Pressure effects on phase stability and melt composition

The sequence of crystallisation of a melt is dependent on variables such as composition, temperature, pressure and fO_2 (e.g. refs.[1–6]). For instance, at low pressures (~0.1 GPa) of fractional crystallisation typical of mid-ocean ridge basalts (MORB), olivine is the first liquidus phase and is follow sequentially at lower [MgO] by plagioclase and clinopyroxene[5]. At higher pressure (>0.8 GPa), the stability of clinopyroxene expands to higher temperatures (i.e., saturates at higher [MgO]), meaning that melts follow a crystallisation trend of olivine followed by clinopyroxene and then plagioclase[1]. Because elements have different compatibilities in these phases, different pressures result in melts following different compositional paths, termed liquid lines of descent, as they progressively cool. Given that the thickness of the crust in oceanic plateau settings (up to 30 km; ref.[7]) is greater than at oceanic spreading ridges (average of ~6.5km; [8]), it is important to constrain how pressure has affected the major element composition of oceanic plateau basalts (OPB) and hence, $[S]_{SULF\text{-}SAT}$ (the concentration of sulfur, [S], at which a melt becomes sulfide-saturated).

To estimate the pressure at which OPB differentiated in the crust, we have modelled fractional crystallisation of the most primitive Kroenke composition (taken as a representative primitive OPB melt; we note that the different OPB suites are not strictly co-genetic) at 0.1, 0.5 and 1.0 GPa using the MELTS algorithm[9]. Each model was run at hydrous conditions (using measured H_2O contents) and oxygen fugacity was initially set to QFM and unbuffered throughout subsequent calculations. Results of this modelling are shown in SI Fig. 1, together with major element data for measured OPB samples, MORB, plume-influenced MORB (PI-MORB) and the ultra-slow spreading Gakkel Ridge[10]. Most OPB lie on or close to a low-pressure (0.1 GPa) fractional crystallisation trend for major elements versus [MgO], including $[FeO_T]$, $[Al_2O_3]$ and $[SiO_2]$ (SI Fig. 1a, b and g, respectively). This modelling is consistent with previous estimates of OPB crustal differentiation pressures which are generally less than 0.4 GPa (e.g., refs.[11–13]). The plot of [CaO] versus [MgO] (SI Fig. 1c) shows the greatest offset between measured data and modelled trends and suggests that OPB underwent high-pressure (1 GPa) differentiation. This offset is because MELTS predicts earlier (i.e., higher [MgO]) clinopyroxene saturation than is typically observed in natural datasets (see ref.[4] for details). Regardless, MELTS modelling demonstrates that plagioclase saturation is delayed to lower temperatures (i.e., lower [MgO]) with increasing pressure. Hence, melts that crystallise at high pressure (~1 GPa) are offset to significantly lower $[FeO_T]$ and therefore lower $[S]_{SULF\text{-}SAT}$, at a given [MgO] than melts crystallising at lower pressures (~0.1 GPa)[14–16].

Because $[S]_{SULF\text{-}SAT}$ is partly dependent on melt composition (mainly $[FeO_T]$; ref.[16]), we have modelled $[S]_{SULF\text{-}SAT}$ isobars for the MELTS models to assess how different liquid lines of descent affect S-systematics (SI Fig. 2). We also include $[S]_{SULF\text{-}SAT}$ isobars for 0.1 and 1.0 GPa experimental data, taken from Yang et al[6] and Elthon and Scarfe[1], respectively. Data for the 1.0 GPa experiments were specifically chosen because they most closely resemble the inferred primitive melt composition of OPB modelled by accumulated fractional melting of fertile peridotite by Herzberg[17]. As the sulfur solubility model of Smythe et al[15] accounts for the Ni and Cu composition of the co-existing sulfide, MELTS and experimental data were given hypothetical Ni and Cu contents. These contents were taken from the measured OPB samples and integrated across each crystallisation interval (~0.1 wt. % MgO for MELTS data) between the highest [MgO] starting composition (high Ni, low Cu) and the lowest [MgO] endpoint (low

Ni, high Cu). The same approach was taken for temperature using Equation 14 from Putirka[18].

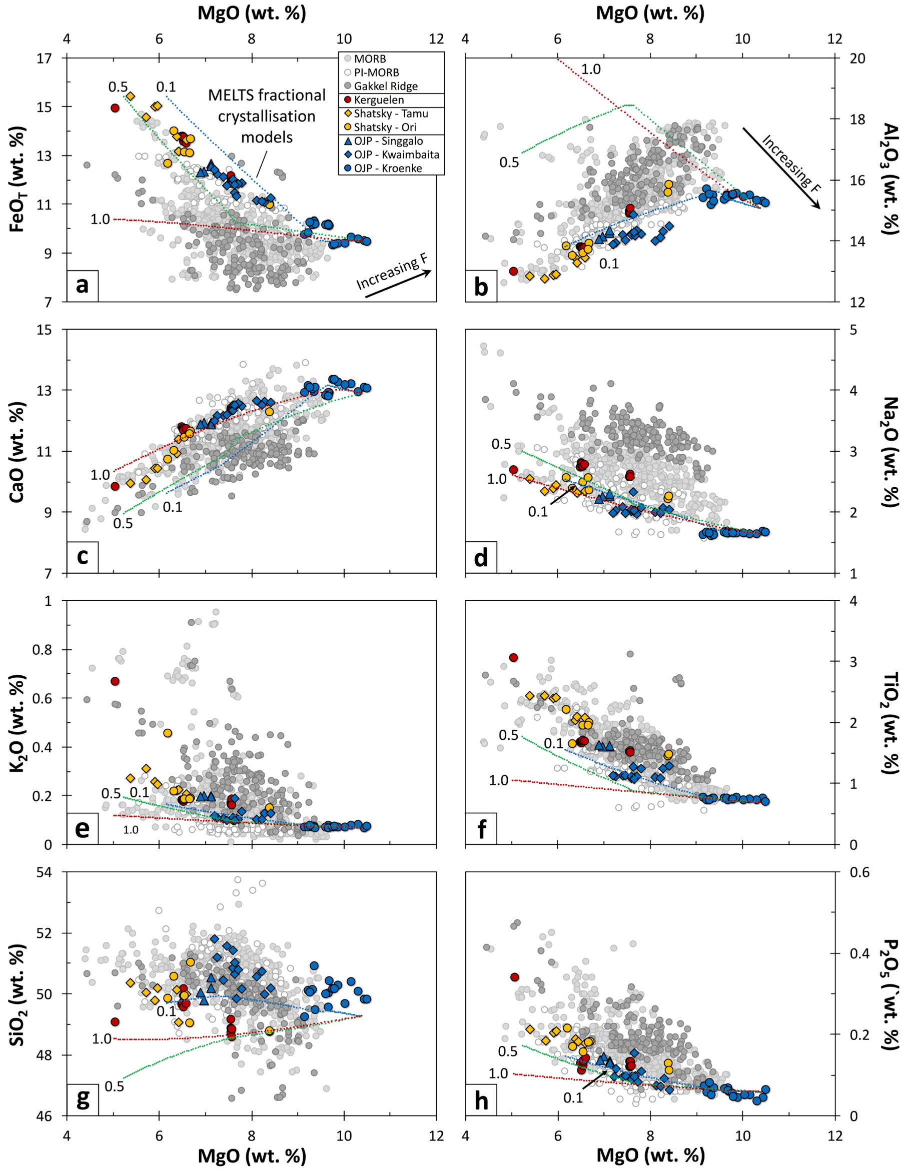

Supplementary Figure 1. Major element systematics during crustal differentiation of OPB and MORB. (**a**) [FeO$_T$], (**b**) [Al_2O_3], (**c**) [CaO], (**d**) [Na_2O], (**e**) [K_2O], (**f**) [TiO_2], (**g**) [SiO_2] and (**h**) [P_2O_5] versus [MgO]. Fractional crystallisation trends for three pressure intervals (0.1, 0.5 and 1.0 GPa) were modelled using the MELTS algorithm[9]. The majority of OPB plot on the low-pressure (0.1 GPa) liquid line of descent indicating that they fractionated at low pressures in the crust following ascent from higher pressures. Notably, both OPB and PI-MORB are offset to higher [FeO$_T$] at a given [MgO] than the ultra-slow spreading Gakkel Ridge and MORB which can be accounted for by higher degrees of partial melting[19]. Similarly, melts which undergo low-pressure differentiation have higher [FeO$_T$] at a given [MgO] because plagioclase saturation shifts to higher temperature (i.e., [MgO]) with decreasing pressure (e.g., ref.[2]). Because [S]$_{SULF\text{-}SAT}$ increases with increasing [FeO$_T$] (e.g., ref.[16]), the slightly higher [FeO$_T$] of OPB relative to most MORB can explain why OPB saturate in sulfide at lower [MgO] than the MORB array, however, these effects are offset by pressure. Data for MORB and the Gakkel Ridge are taken from Jenner and O'Neill[20] and Gale et al[10], respectively. Vectors of increasing F are taken from Wasylenki et al[19].

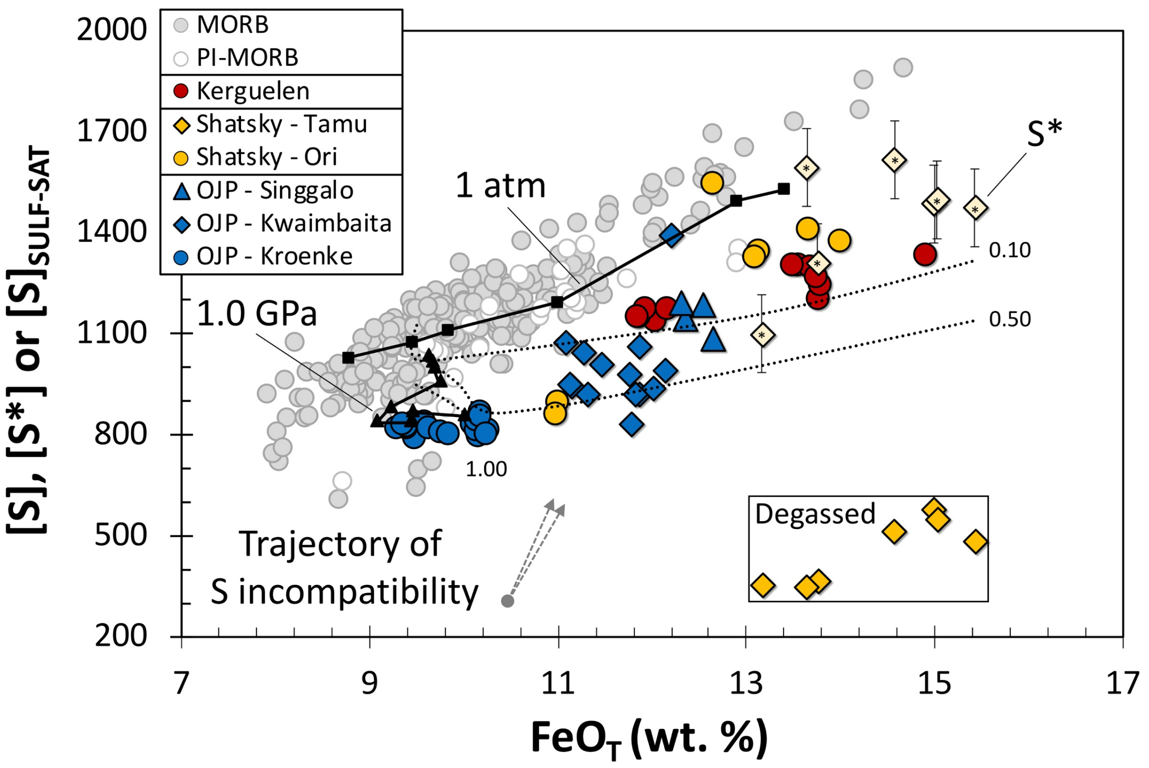

Supplementary Figure 2. Sulfur solubility modelling of MELTS and experimental data. $[S]_{SULF\text{-}SAT}$ isobars are modelled for MELTS and experimental data to demonstrate how different liquid lines of descent (i.e., different pressures of crustal differentiation) can affect S-solubility in basaltic melts. The progressive offset to low [S] with increasing pressure of the MELTS isobars reflects the delayed onset of plagioclase saturation with increasing pressure, and hence lower $[FeO_T]$ at a given [MgO]. The low-pressure (0.1 GPa) experimental isobar (ref.[6]) closely resembles MORB, which show increasing [S] with increasing $[FeO_T]$. By contrast, $[S]_{SULF\text{-}SAT}$ at 1.0 GPa (ref.[1]) first declines with little variation in $[FeO_T]$. This is caused by a prolonged 'olivine-only' crystallisation interval from ~16 to ~10 wt. % [MgO] prior to the onset of clinopyroxene and then plagioclase saturation. Hence, both MELTS data and high-pressure experimental data predict that high-pressure olivine crystallisation can lower $[S]_{SULF\text{-}SAT}$ prior to ascent and differentiation at lower pressure. Discrepancies in the onset of clinopyroxene crystallisation between MELTS and natural samples limit the application of MELTS to studying S-systematics during high-pressure differentiation (e.g., crystallisation of melts lower in the crust prior to ascent). Data for MORB are taken from Jenner and O'Neill[20]. Error bars on [S*] (see main text) are given as the propagated 1SD error on repeat analysis of the Se standard.

The 1 atm experimental isobar closely overlaps with the MORB array, which is consistent with previous suggestions that MORB are sulfide-saturated during low-pressure crustal differentiation (e.g., refs.[21,22]). At 1.0 GPa, olivine-only crystallisation increases $[Al_2O_3]$ while $[FeO_T]$ remains near-invariant. This has the effect of decreasing $[S]_{SULF\text{-}SAT}$ between 16 and 10 wt. % MgO. However, as the Kroenke basalts show no evidence for sulfide segregation (i.e., Pt and Au are not depleted relative to primitive MORB; SI Fig. 3) it is likely that they were sulfide-under-saturated prior to ascent through the crust and eruption.

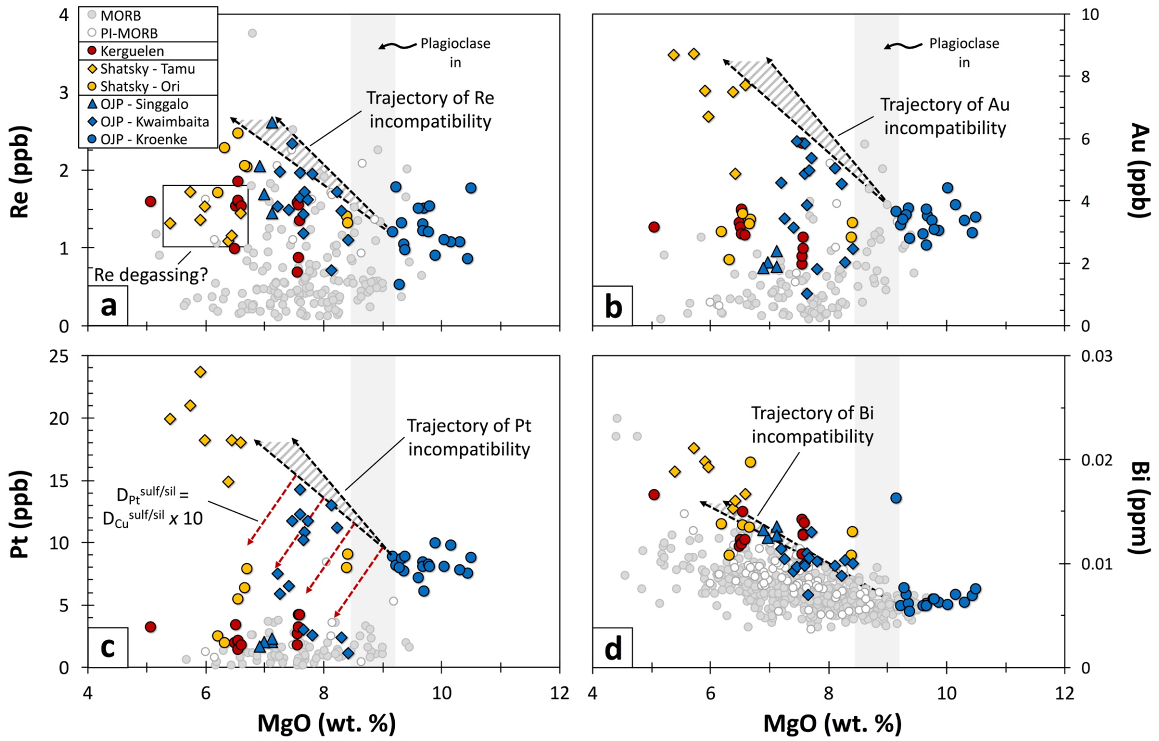

Supplementary Figure 3. Siderophile element variations during crustal differentiation of OPB and MORB. (**a**) [Re], (**b**) [Au], (**c**) [Pt] and (**d**) [Bi] versus [MgO]. Qualitative trajectories for increasing [Re], [Au], [Pt] and [Bi] in sulfide-under-saturated melts (dashed triangle) are modelled from the slope of Rb (bulk-D of ~0), which is assumed to be perfectly incompatible during crustal differentiation (see main text). As with Cu and Ag, some of the Kwaimbaita, Ori Massif and Kerguelen samples plot below these 'D=0' trajectories, indicating that these melts are sulfide-saturated. Unlike [Pt] and [Au] versus [MgO], the contents of Re in the Tamu Massif samples are offset from the 'D=0' line suggesting that Re is at least partially degassed[23]. Lines approximating sulfide fractionation (red dashed lines) are shown for [Pt] versus [MgO]. Because Pt is highly compatible in sulfide ($D^{sulf/sil}$ >10,000; ref.[24]), slopes for Pt in MORB have a large error associated with them. Hence, we have modelled these lines using the slopes of Cu from Jenner[22] as $D_{Pt} = D_{Cu}$ *x* 10. Notably, the steepness of the sulfide fractionation lines suggests that the offset in [Pt] between primitive (high-[MgO]) MORB and OPB (Kroenke) can readily be explained by sulfide-saturation, as is shown in MORB, and does not require anomalous depletions of Pt in the MORB-source mantle. High [PGE] in primitive melts are commonly used as evidence to suggest that sulfide is exhausted from the OPB mantle source[25] (see main text). Note: siderophile element concentrations are given as information values only because contents lie close to the limit of detection. Data for MORB are taken from Jenner and O'Neill[20].

At all pressure intervals modelled using MELTS data, olivine ± clinopyroxene crystallisation at low [FeO_T] decreases $[S]_{SULF\text{-}SAT}$. The magnitude of this decrease with increasing pressure reflects the progressive delay in plagioclase saturation to lower [MgO]. Thus, in addition to the direct pressure effects on $[S]_{SULF\text{-}SAT}$, melts which undergo high-pressure differentiation have a lower $[S]_{SULF\text{-}SAT}$ relative to melts crystallising at low pressure (0.1 versus 1.0 GPa MELTS isobars in SI Fig. 2). Although the MELTS isobars and the OPB datasets appear to be in good agreement (SI Fig. 2), MELTS isobars are offset to lower [S] at a

given [FeO_T] compared to the 0.1 GPa experimental model. This offset is because MELTS does not sufficiently reproduce the behaviour of CaO during crystallisation (SI Fig. 1). Thermodynamic models also do not account for the effects of magma recharge which are implied by the modelled slopes of MORB and potentially OPB (see ref.[26]). Because of these uncertainties, we favour $[S]_{SULF\text{-}SAT}$ isobars modelled from the measured OPB glass compositions. However, we stress that, with the current models available, there is no one unique solution to modelling $[S]_{SULF\text{-}SAT}$ in basaltic melts.

Sulfide globules were routinely encountered during laser ablation of the OPB glasses. Such scans were rejected and the samples re-analysed on a fresh spot. Accidental ablation of sulfide is apparent as a concomitant increase in the signal intensity of highly chalcophile elements (e.g., Cu and Ag, which have a $D^{sulf/sil}$ of ~1000; ref[22]) while the signal intensity of significantly less chalcophile elements (e.g., Sn, which has a $D^{sulf/sil}$ of ~8; ref.[22]) remains constant throughout the ablation interval. An example of accidental ablation of a sulfide globule is given for Kerguelen sample IODP-2 in SI Fig. 4.

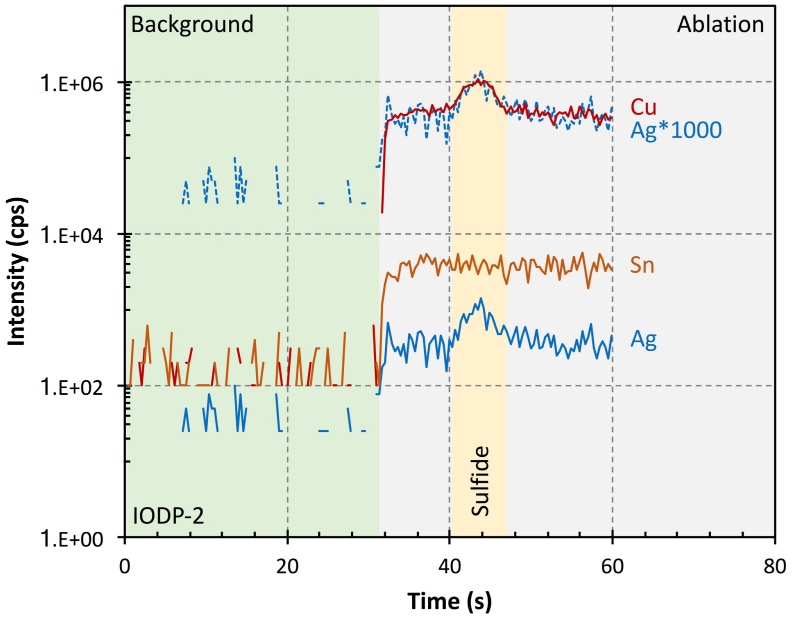

Supplementary Figure 4. LA-ICP-MS scan of Kerguelen sample IODP-2 demonstrating accidental sulfide ablation. The accidental ablation of sulfides is apparent as a concomitant increase in the intensity (counts per second, cps) of the LA-ICP-MS signal for the highly-chalcophile elements (e.g., Cu and Ag) in contrast to the signal intensity of elements which have a low $D^{sulf/sil}$ (e.g., Sn). To prevent biasing the composition of the glass sample, such scans were rejected and the glass re-analysed on a fresh surface. The small size of the sulfide blebs limited the possibility of identifying them visually prior to analysis

Mantle controls on sulfide-saturation

In addition to the effects of pressure on melt trajectories during fractional crystallisation, the compositions of partial melts in equilibrium with their mantle source are also dependent on pressure[19,27,28]. For example, the OPB magmas have higher [FeO_T] at a

given [MgO] compared to the Gakkel Ridge and lie at the high end of the MORB array (SI Fig. 1). These offsets are consistent with inferred differences in the degree of melting (F) between these settings as, for a given pressure, [FeO_T] of partial melts increases with increasing F. Thus, aside from the pressure effects on $[S]_{SULF-SAT}$ during partial melting, melts which formed at higher F, at a given pressure, will have higher $[S]_{SULF-SAT}$. However, during adiabatic decompression (i.e., in a polybaric melting regime), there is a decrease in [FeO_T] with increasing F because of pressure effects on the composition of the partial melt in equilibrium with its mantle source (see ref.[29] for further details). This effect complicates comparison of melts erupting across different geological settings and consequently, melts which formed at different conditions in the mantle. Furthermore, despite higher [FeO_T] (which should increase $[S]_{SULF-SAT}$; see ref.[16]), OPB are offset to lower $[S]_{SULF-SAT}$ at a given [FeO_T] than MORB (SI Fig. 2). This suggests that the direct effects of pressure, and other compositional variables (e.g., $[S]_{SULF-SAT}$ decreases with increasing Cu and Ni content), offset the effects of increased [FeO_T] caused by higher degrees of partial melting. In summary, the ambiguity regarding 1) the behaviour of FeO_T during partial melting, 2) differences in the temperature and pressure during partial melting of the MORB and OPB source mantle and 3) the S budget of the Earth (i.e., the degree of melting required to exhaust sulfide from the mantle source) make estimating $[S]_{SULF-SAT}$ during mantle processes extremely challenging. There is also considerable disagreement between the estimated $[S]_{SULF-SAT}$ depending on what models are used (e.g., refs.[15,30,31]; see Ding and Dasgupta[32] for further details).

Lastly, we consider links between mantle source composition (fertility), depth of melting and sulfide-saturation during differentiation of OPB. La/Sm and Gd/Yb (SI Fig. 5a and 5b) are commonly used to place constraints on mantle source composition and mineralogy, degree of partial melting and/or pressure of partial melting (e.g., ref.[33]). For example, mantle-derived melts with low primitive-mantle-normalised La/Sm ($(La/Sm)_N$ <1; see ref.[33] and references therein) are considered to be derived from more depleted mantle sources than melts with higher $(La/Sm)_N$. Similarly, because Yb is more compatible in garnet than Gd[34], high Gd/Yb may indicate that garnet is a residual phase in the mantle source during partial melting, and consequently that melts were derived at greater depths in the garnet stability field than melts with lower Gd/Yb[35].

The La/Sm of the various OPB suites, with the exception of the most-evolved Kerguelen and Ori-Massif samples, are similar and show considerably less variability at a given [MgO] compared to MORB (SI Fig. 5a). This indicates that the basalts formed by similar degrees of partial melting and/or that the fertility of the mantle source regions of each of the plateaus was comparable. This is consistent with major element trends (see SI Fig. 1): the OPB array show limited variations in various major elements at a given [MgO] compared to the highly variable MORB array. There is no correlation between La/Sm and the stage of differentiation (i.e., [MgO]) at which the melts from different plateaus reach sulfide-saturation. Basalts from the Shatsky Rise have slightly elevated Gd/Yb relative to other OPB suites, which might indicate the presence of residual garnet in their mantle source and consequently, higher pressures of melting (SI Fig. 5b). However, as with La/Sm, there is no correlation between Gd/Yb and the stage of differentiation at which the OPB suites saturate in sulfide. For example, samples from the Ori Massif and the Kwaimbaita (Ontong Java Plateau) have different La/Sm and Gd/Yb yet reach sulfide-saturation across a similar [MgO] range (see Fig. 1 of main text). Importantly, these ratios also cannot explain why MORB reach sulfide-saturation at higher [MgO] compared to OPB.

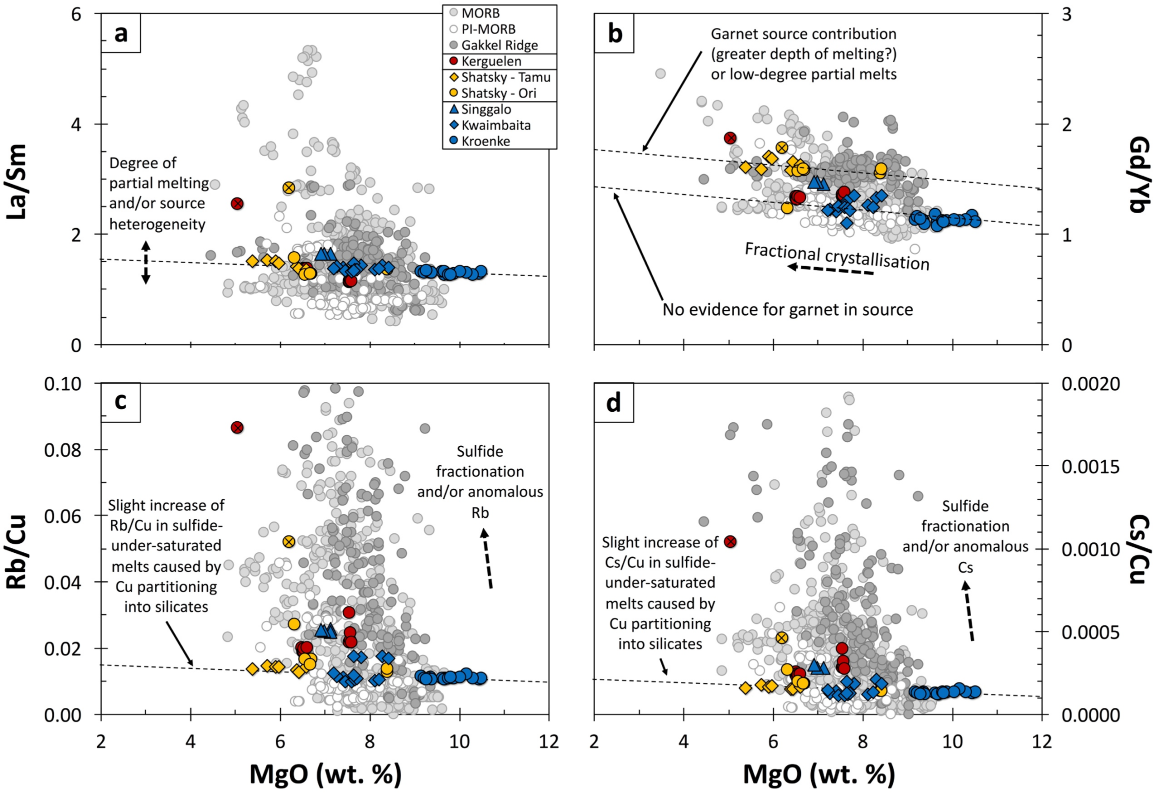

Supplementary Figure 5. La/Sm, Gd/Yb, Rb/Cu and Cs/Cu systematics during crustal differentiation of OPB and MORB. **a, b,** La/Sm and Gd/Yb are commonly used as proxies to place constraints on mantle composition and mineralogy, degree of partial melting and/or the pressure of partial melting during the petrogenesis of mantle-derived melts (e.g., ref.[33]). Despite higher Gd/Yb of Shatsky Rise magmas compared to other OPB suites and subtly different La/Sm, these ratios cannot explain why OPB saturate in sulfide at different [MgO] or importantly, why MORB saturate at higher [MgO] compared to OPB. **c, d,** Rb/Cu and Cs/Cu show a slight increase with decreasing [MgO] in sulfide-under-saturated melts because the bulk-D of Cu is controlled by partitioning into silicate minerals in the absence of sulfide. At sulfide-saturation, Cu becomes compatible in sulfide and consequently the Rb/Cu increases significantly as is shown by MORB and some Kerguelen, Ori Massif and Kwaimbaita samples. Notably, however, the variability of incompatible elements (e.g., Rb and Cs), which are sensitive to subtle changes in the degree and/or depth of partial melting and fertility, limit the usefulness of these ratios for constraining the [MgO] at which melts become sulfide-saturated (samples which are outliers on incompatible element (e.g., Rb and Cs) versus [MgO] plots are marked by crosses). MORB data are taken from Jenner and O'Neill[20].

Our slope modelling indicates that the bulk-D of Cu is comparable (i.e., assuming D=0 behaviour) or slightly higher (i.e., assuming Cu partitioning into silicate minerals only, see above) than the bulk-D of Rb during crustal differentiation of a sulfide-under-saturated melt. By contrast, when a melt becomes sulfide-saturated, the bulk-D of Cu increases significantly (i.e., Cu has a positive slope in sulfide-saturated MORB). This means that highly incompatible element-to-Cu ratios (e.g., Rb/Cu and Cs/Cu; SI Fig. 5c and 5d) can be used to place additional constraints on sulfide-systematics during partial melting and crustal differentiation. Samples with elevated Rb/Cu and Cs/Cu also have anomalously high La/Sm indicating that their source

regions might have been more enriched (i.e., more incompatible elements and potentially S), and therefore should have achieved sulfide-saturation earlier (i.e., at higher [MgO]) than other OPB samples with lower La/Sm. However, higher [MgO] Kerguelen and Ori Massif samples with lower La/Sm, comparable to other OPB suites, appear to achieve sulfide-saturation at the highest [MgO] suggesting that source fertility has limited influence on the stage at which the melts saturated in sulfide during crustal differentiation. Furthermore, while the Rb/Cu and Cs/Cu ratios should increase at sulfide-saturation (Cu is compatible in sulfide whereas Rb and Cs remain incompatible in the melt), as is shown by MORB, the variability of the highly incompatible elements in the different OPB suites limits the application of this proxy to determining the stage of differentiation (i.e., [MgO]) at which the various OPB suites saturate in sulfide. For example, in the presence of residual sulfide, [Cu] of the melt increases with increasing degrees of partial melting, whereas [Rb] of the melt decreases with increasing degrees of partial melting. However, many of the samples from the Tamu and Ori Massif have comparable Rb/Cu.

Supplementary Methods

Slope calculations and bulk-partition coefficients

Differences in $d(\log([M])/d([MgO]))$, termed 'slopes', can be used to compare the relative bulk-partition coefficients (bulk-D) of trace elements in a suite of magmas that follow a similar liquid line of descent. Extensive description of this method, including the necessary equations and error estimations, are given by O'Neill and Jenner[26] and Jenner[22]. Here, we give an example of slope calculations for OPB and MORB (SI Fig. 6)

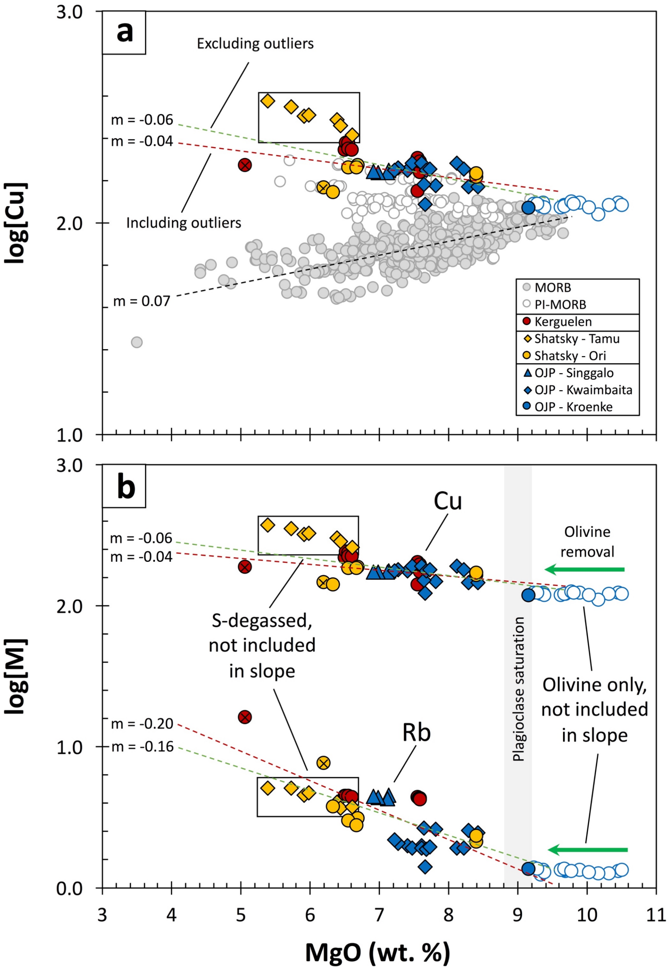

Supplementary Figure 6 (above). Example slope calculations for OPB and MORB. **a**, log([Cu]) versus [MgO] for OPB and MORB showing the differences between positive and negative slopes. The more positive a slope, the greater the relative bulk-distribution coefficient of the element in the crystallising phase assemblage during crustal differentiation. Note that plume-influenced MORB are excluded from the calculation of MORB slopes because these often show subtly different trace element compositions to global MORB. **b**, log([Cu]) and log([Rb]) versus [MgO] for OPB and MORB. These elements have different bulk-distribution coefficients during crustal differentiation which can be compared qualitatively by calculating the slope of log([M]) versus [MgO]. To allow comparison between the slopes of MORB and OPB, OPB slopes are only calculated for plagioclase-saturated samples and the most evolved Kroenke sample (filled blue circle). Chalcophile element-enriched Tamu Massif samples, which we suggest may have resorbed sulfide during S-degassing, are also excluded from the calculations to avoid biasing the slope of chalcophile elements. Because each plateau show subtly different incompatible element (e.g., Rb, Cs and Th) contents versus [MgO], we calculate two slopes for each element including and excluding outliers on incompatible element versus [MgO] plots (two outlier samples marked by crosses). These slopes are within error for elements with a bulk-D greater than La. Using both slopes for Rb (i.e., including and excluding outliers), which we assume to have a bulk-D of ~0, provides a best-estimate of the behaviour of the CSE in sulfide-under-saturated melt. Data for MORB are taken from Jenner and O'Neill[20].

SI Fig. 6a is a plot of log([Cu]) versus [MgO] showing data for MORB, PI-MORB and OPB (note: PI-MORB data are not included in MORB slope calculations) and the resultant slopes (gradient of log([M]) versus [MgO]). Because MORB are plagioclase-saturated, direct comparison between the slopes of MORB and OPB can only be made if high-[MgO] 'olivine-only' samples (i.e., melts where plagioclase is not on the liquidus) are excluded from slope calculations (omitted samples from OPB slopes shown, for clarity, on SI Fig. 6b). Similarly, because the chalcophile element systematics of Tamu Massif samples suggest that they may have resorbed sulfide, we exclude these samples from the OPB slope calculations to avoid biasing the slopes of the chalcophile elements. To provide an upper and lower limit of possible bulk-D=0 behaviour in the melts, slopes are calculated for the remaining samples with and without outliers in incompatible element (e.g., Rb, Cs and Th) versus [MgO] plots (two outlier samples with high log([Rb]) at low [MgO] marked by cross on SI Fig. 6b). This method provides a best-estimate of the possible range in bulk-partitioning of each element in the OPB 'array'.

The calculated slope of each element represents a complex interplay between mineral-melt partition coefficients, melt pressure and temperature, fO_2, magma chamber recharge and depletion and the modal proportions of the crystallising phase assemblage[26]. As with MORB, the slope of Rb in OPB has the lowest value relative to all other trace elements (see Fig. 5 of main text). This behaviour led O'Neill and Jenner[26] to suggest that Rb has a bulk-D of effectively ~0 during crustal differentiation. Consequently, we use the slope of Rb to model 'incompatible behaviour' of chalcophile elements at sulfide-under-saturation (grey dashed triangles in SI Fig. 3 and Fig. 1 of main text). In addition, because Cu has a comparable $D^{min/melt}$ to Nd in silicate minerals), we use the slope of Nd to constrain the behaviour of Cu assuming that the melt is sulfide-under-saturated and silicate minerals are the only fractionating phases during crustal differentiation (i.e., to provide a more realistic estimate of the behaviour of Cu in a sulfide-under-saturated OPB melt; grey field in Fig. 1a of main text). Importantly, the measured slopes of Cu (i.e., including and excluding outliers, see above) are less negative than the slopes of Rb and Nd which suggests that the bulk-partitioning of Cu is controlled by both silicate mineral and sulfide fractionation.

Supplementary References

1. Elthon, D. & Scarfe, C. M. High-pressure phase equilibria of a high-magnesia basalt and the genesis of primary oceanic basalts. *Am. Mineral.* **69,** 1–15 (1984).
2. Husen, A., Almeev, R. R. & Holtz, F. The effect of H_2O and pressure on multiple saturation and liquid lines of descent in basalt from the Shatsky Rise. *J. Petrol.* **57,** 309–344 (2016).
3. Villiger, S., Ulmer, P., Müntener, O. & Thompson, A. B. The liquid line of descent of anhydrous, mantle-derived, tholeiitic liquids by fractional and equilibrium crystallization - An experimental study at 1·0 GPa. *J. Petrol.* **45,** 2369–2388 (2004).
4. Voigt, M., Coogan, L. A. & von der Handt, A. Experimental investigation of the stability of clinopyroxene in mid-ocean ridge basalts: The role of Cr and Ca/Al. *Lithos* **274–275,** 240–253 (2017).
5. Whitaker, M. L., Nekvasil, H., Lindsley, D. H. & Difrancesco, N. J. The role of pressure in producing compositional diversity in intraplate basaltic magmas. *J. Petrol.* **48,** 365–393 (2007).
6. Yang, H., Kinzler, R. J. & Grove, T. L. Experiments and models of anhydrous, basaltic olivine-plagioclase-augite saturated melts. *Contrib. to Mineral. Petrol.* **124,** 1–18 (1996).
7. Gladczenko, T. P., Coffin, M. F. & Eldholm, O. Crustal structure of the Ontong Java Plateau: Modeling of new gravity and existing seismic data. *J. Geophys. Res.* **102,** 22,711-22,729 (1997).
8. White, W. M. Composition of the oceanic crust. *Treatise on Geochemistry* **4,** 457–496 (2014).
9. Gualda, G. A. R., Ghiorso, M. S., Lemons, R. V. & Carley, T. L. Rhyolite-MELTS: A modified calibration of MELTS optimized for silica-rich, fluid-bearing magmatic systems. *J. Petrol.* **53,** 875–890 (2012).
10. Gale, A., Dalton, C. A., Langmuir, C. H., Su, Y. & Schilling, J. G. The mean composition of ocean ridge basalts. *Geochemistry, Geophys. Geosystems* **14,** 489–518 (2013).
11. Husen, A. *et al.* Geothermobarometry of basaltic glasses from the Tamu Massif, Shatsky Rise oceanic plateau. *Geochemistry, Geophys. Geosystems* **14,** 3908–3928 (2013).
12. Sano, T. & Yamashita, S. Experimental petrology of basement lavas from Ocean Drilling Program Leg 192: Implications for differentiation processes in Ontong Java Plateau magmas. *Geol. Soc. London, Spec. Publ.* **229,** 185–218 (2004).
13. Wallace, P. J. Volatiles in submarine basaltic glasses from the northern Kerguelen Plateau (ODP Site 1140): Implications for source region compositions, magmatic processes, and plateau subsidence. *J. Petrol.* **43,** 1311–1326 (2002).
14. Mavrogenes, J. A. & O'Neill, H. St. C. The relative effects of pressure, temperature and oxygen fugacity on the solubility of sulfide in mafic magmas. *Geochim. Cosmochim. Acta* **63,** 1173–1180 (1999).
15. Smythe, D. J., Wood, B. J. & Kiseeva, E. S. The S content of silicate melts at sulfide saturation: New experiments and a model incorporating the effects of sulfide composition. *Am. Mineral.* **102,** 795–803 (2017).
16. Wykes, J. L., O'Neill, H. St. C. & Mavrogenes, J. A. The effect of FeO on the sulfur content at sulfide saturation (SCSS) and the selenium content at selenide saturation of silicate melts. *J. Petrol.* **56,** 1407–1424 (2014).

17. Herzberg, C. Partial melting below the Ontong Java Plateau. *Geol. Soc. London, Spec. Publ.* **229,** 179–183 (2004).
18. Putirka, K. D. Thermometers and barometers for volcanic systems. *Rev. Mineral. Geochemistry* **69,** 61–120 (2008).
19. Wasylenki, L. E., Baker, M. B., Kent, A. J. R. & Stolper, E. M. Near-solidus melting of the shallow upper mantle: Partial melting experiments on depleted peridotite. *J. Petrol.* **44,** 1163–1191 (2003).
20. Jenner, F. E. & O'Neill, H. St. C. Analysis of 60 elements in 616 ocean floor basaltic glasses. *Geochemistry, Geophys. Geosystems* **13,** Q02005 (2012).
21. Francis, R. D. Sulfide globules in mid-ocean ridge basalts (MORB), and the effect of oxygen abundance in Fe-S-O liquids on the ability of those liquids to partition metals from MORB and komatiite magmas. *Chem. Geol.* **85,** 199–213 (1990).
22. Jenner, F. E. Cumulate causes for the low contents of sulfide-loving elements in the continental crust. *Nat. Geosci.* **10,** 524–529 (2017).
23. Lassiter, J. C. Rhenium volatility in subaerial lavas: Constraints from subaerial and submarine portions of the HSDP-2 Mauna Kea drillcore. *Earth Planet. Sci. Lett.* **214,** 311–325 (2003).
24. Patten, C., Barnes, S. J., Mathez, E. A. & Jenner, F. E. Partition coefficients of chalcophile elements between sulfide and silicate melts and the early crystallization history of sulfide liquid: LA-ICP-MS analysis of MORB sulfide droplets. *Chem. Geol.* **358,** 170–188 (2013).
25. Ely, J. C. & Neal, C. R. Using platinum-group elements to investigate the origin of the Ontong Java Plateau, SW Pacific. *Chem. Geol.* **196,** 235–257 (2003).
26. O'Neill, H. St. C. & Jenner, F. E. The global pattern of trace-element distributions in ocean floor basalts. *Nature* **491,** 698–704 (2012).
27. Hirose, K. & Kushiro, I. The effect of melt segregation on polybaric mantle melting: Estimation from the incremental melting experiments. *Phys. Earth Planet. Inter.* **107,** 111–118 (1998).
28. Hirschmann, M. M., Ghiorso, M. S. & Stolper, E. M. Calculation of peridotite partial melting from thermodynamic models of minerals and melts. II. Isobaric variations in melts near the solidus and owing to variable source composition. *J. Petrol.* **40,** 297–313 (1999).
29. Asimow, P.D., Hirschmann, M.M. & Stolper, E.M. Calculation of peridotite partial melting from thermodynamic models of minerals and melts, IV. Adiabatic decompression and the composition and mean properties of mid-ocean ridge basalts. *J. Petrol.* **42,** 963-998 (2001).
30. Fortin, M. A., Riddle, J., Desjardins-Langlais, Y. & Baker, D. R. The effect of water on the sulfur concentration at sulfide saturation (SCSS) in natural melts. *Geochim. Cosmochim. Acta* **160,** 100–116 (2015).
31. Li, C. & Ripley, E. M. Empirical equations to predict the sulfur content of mafic magmas at sulfide saturation and applications to magmatic sulfide deposits. *Miner. Depos.* **40,** 218–230 (2005).
32. Ding, S. & Dasgupta, R. The fate of sulfide during decompression melting of peridotite – Implications for sulfur inventory of the MORB-source depleted upper mantle. *Earth Planet. Sci. Lett.* **459,** 183–195 (2017).
33. Hofmann, A. W. Sampling mantle heterogeneity through oceanic basalts: Isotopes and trace elements. *Treatise on Geochemistry* **2,** 1–44 (2007).

34. Nicholls, I. A. & Harris, K. L. Experimental rare earth element partition coefficients for garnet, clinopyroxene and amphibole coexisting with andesitic and basaltic liquids. *Geochim. Cosmochim. Acta* **44,** 287–308 (1980).
35. O'Neill, H. St. C. The smoothness and shapes of chondrite-normalized rare earth element patterns in basalts. *J. Petrol.* **57,** 1463–1508 (2016).
