# Supplementary material for: Sulfide resorption during crustal ascent and degassing of oceanic plateau basalts
Source: Nat Commun. 2019 Jan 8;10:82. doi: 10.1038/s41467-018-08001-3 (PMC6325133; doi:10.1038/s41467-018-08001-3)
Supplement: Supplementary file 3 — Description of Additional Supplementary Files [file 41467_2018_8001_MOESM3_ESM.pdf]

1. Supplementary table 1 – Compositional data for BCR-2G
2. Supplementary table 2 – Compositional data for the NWLSC standards
3. Supplementary table 3 – Major, trace and volatile element compositions of analysed oceanic plateau basalt samples
4. Supplementary table 4 – Updated compositional data for selected elements re-analysed in this study alongside the MORB data of Jenner and O'Neill (2012)
5. Supplementary table 5 – Slopes and parental-MORB values from Jenner (2017) and updated data for Se, Mo and Sn
6. Supplementary table 6 – Average melt chalcophile and siderophile element compositions and mantle source estimations for OPB and parental-MORB
